# Isolated tuberculosis liver abscess in an immunocompetent patient

**DOI:** 10.1002/ccr3.5049

**Published:** 2021-11-09

**Authors:** Yazan M. Sallam, Ramsey Gasim, Mohammad N. Kloub, Nabeel Mohammad Qassem

**Affiliations:** ^1^ Internal Medicine Department Hamad Medical Corporation Doha Qatar

**Keywords:** extrapulmonary tuberculosis, liver abscess, tuberculous

## Abstract

Isolated TLA is an extremely rare condition, but should always be considered in a patient presented with liver abscess, especially from an endemic area. Diagnosis depends on histological identification, with treatment being quadruple therapy.

## Graphical abstract

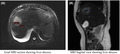

## 1 INTRODUCTION

Tuberculous (TB) liver involvement is a rare extrapulmonary manifestation of TB and is usually found as a secondary involvement to TB of the lung. Patients usually present with vague symptoms and are nonspecific laboratory findings. Imaging modalities do not help differentiate between pyogenic or amoebic abscess from TB liver abscess (TLA). Histopathological examination is essential for the diagnosis as it shows granuloma formation with or without caseation in most cases, with PCR assays aiding in the diagnosis as it provides a high sensitivity rate. Definite treatment involves conventional TB treatment for a period lasting up to 1 year. In this article, we are presenting a case of an isolated TLA in an immunocompetent patient.

Globally, an estimated 10.0 million people fell ill with (TB) in 2019. TB is a communicable disease that is a major health concern around the world, one of the top 10 causes of death globally, and the leading cause of death from an infectious agent.^1^ TLA without active pulmonary or military TB is a rare entity.^2^ Patients usually present with nonspecific symptoms, which makes it difficult to diagnose. Here, we present a case of an isolated TLA in an immunocompetent patient, in which the patient had symptoms of abdominal pain, vomiting, and fever, with no specific findings to suggest TLA on radiological imaging.

## 2 CASE PRESENTATION

A 62‐year‐old male patient presented to our hospital, complaining of a fever of 10‐day duration.

This patient is a known case of CKD stage 5, on regular follow‐ups, was doing relatively fine when he started having fever for 10 days before presenting to our hospital, and the fever was mainly at night, documented at about 38 degrees, associated with chills and rigors, and occasional vomiting.

On physical examination, except for right upper quadrant tenderness, he had no other physical exam findings.

Laboratories showed: hemoglobin 9.0 gm/dl, white blood cells 24,000/mm^3^ (neutrophils 88%), platelets count 322,000, blood urea nitrogen 39 mmol/L(high), creatinine 567 umol/L (this is his baseline kidney function), C‐reactive protein 136 mg/L (high), total bilirubin 21 umol/L (normal range up to 22), elevated liver enzymes, and alkaline phosphatase 514 U/L (normal range up to 129).

The patient was investigated for hepatitis B, hepatitis C, and HIV, all were negative, chest X‐ray was normal, and routine microscopic examination of stool showed no cyst or ova; amoebic serology was negative.

**FIGURE 1:**
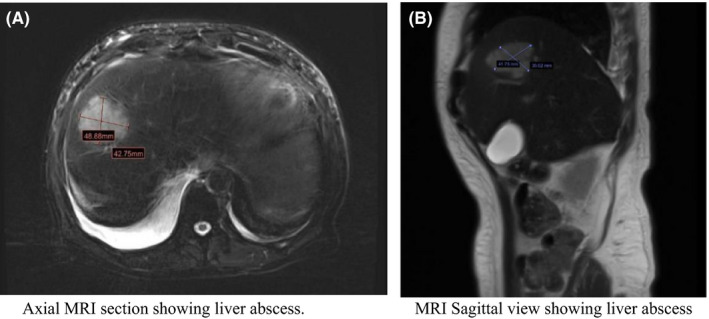
(A) Axial MRI section showing liver abscess. (B) MRI Sagittal view showing liver abscess

US abdomen was done, which showed Ill‐defined heterogeneous structure noted at the right lobe liver, so MRI abdomen was done for the patient, without contrast, which showed focal hepatic lesion in segment VIII/IVb (4.8 cm in maximal dimension), enlarged abdominal lymph nodes, including portocaval lymph nodes which are compressing the mid‐CBD and causing upstream biliary dilatation (Figure 1).

US‐guided aspiration was attempted, but the content was very thick that nothing was aspirated, so eight liver biopsies were taken from the right liver lobe lesion using a coaxial 18/16 G biopsy system.

The biopsies were sent for Ziehl‐Neelsen (ZN) stain, acid‐fast bacilli culture, polymerase chain reaction (PCR) for TB, and other routine microbiological investigations alongside histopathology.

TB PCR came positive the next day, and histopathology showed necrotizing granulomatous inflammation. Diagnosis of TLA was made, and the patient was started on anti‐TB medications (isoniazid 300 mg daily, rifampin 600 mg daily, pyrazinamide 1750 mg Q48 hours, and ethambutol 800 mg Q48 hours). After 3 days of starting the treatment, the patient started to improve clinically, evidenced by the absence of fever, gradual improvement of the abdominal pain and vomiting, and decrease in inflammatory markers.

The patient was discharged after 1 week from starting treatment, with regular follow‐ups scheduled, he came back to the infectious disease clinic after 1 month, with complete resolution of GI symptoms, and no recurrent fever, repeat US was not done as the patient was clinically improving.

## 3 DISCUSSION

Tuberculous infection remains one of the leading causes of death, especially in developing countries.^3^ It is extremely important for clinicians to understand the nature of the disease and its wide variety of presentations, in addition to having a high index of suspension when dealing with patients in areas where TB remains an endemic infection.

In extrapulmonary TB, liver tuberculosis has been described as a rare entity, but not an exceptional one. Isolated TLA is an extremely rare condition, The incidence of which was found to be 0.34%.^4^ It is often misdiagnosed as a pyogenic or amoebic abscess.^5^ Most of the cases in the literature occur alongside miliary TB of the lungs, which spread to the liver by hematogenous spread.^6^

The diagnosis of TLA has always been challenging as the symptoms of this condition are not specific.^7^ Patients usually present with constitutional symptoms such as fever, anorexia, and weight loss. As there are no specific symptoms, signs, or laboratory investigations for TLA, diagnosis depends on a high index of suspicion, especially in patients coming from endemic areas for TB. Radiological imaging modalities are usually not helpful in differentiating between pyogenic, amoebic, or TB liver abscess.^8^ In our case, our patient came with vague symptoms, which manifested mainly as fever and abdominal pain.

The diagnosis of TLA requires the use of ZN stains, acid‐fast bacilli culture, and PCR on the specimen collected.^9^ In liver biopsies, granuloma formation can be seen in around 80%–100% of cases and caseation in up to 83%.^10^ PCR assays are positive in up to 88% of TLA cases.^11^ No specific laboratory investigations can help diagnose TLA, with previous studies showing elevated alkaline phosphatase as the most frequent finding.^12^

Medical treatment for TB is a debatable subject, with most centers recommending treatment with quadruple therapy for 1 year.^13^ In our case, treatment with anti‐TB medication was started for the patient and he started showing improvement within 1 week of starting the medications, and he was seen 1 month after discharge with complete resolution of the fever and his previous symptoms.

## 4 CONCLUSION

Isolated TLA is an extremely rare condition, but should always be considered in a patient presented with liver abscess, especially from an endemic area. There are no specific symptoms or signs for TLA, and radiological investigations usually do not help differentiate between TLA and other causes of liver abscess. Treatment with quadruple therapy is the mainstay for treatment, and the prognosis is usually good.

## CONFLICTS OF INTEREST

The authors report no conflicts of interest in this work.

## AUTHOR CONTRIBUTIONS

All authors contributed equally in writing and editing.

## ETHICAL APPROVAL

Case approved by HMC Medical Research Center.

## CONSENT

Written informed consent was obtained from the patient to allow the publication of information including images.

## Data Availability

Data sharing is not applicable to this article as no new data were created or analyzed in this study.
